# Alexithymia and Bipolar Disorder: Virtual Reality Could Be a Useful Tool for the Treatment and Prevention of These Conditions in People with a Physical Comorbidity

**DOI:** 10.3390/jcm13206206

**Published:** 2024-10-18

**Authors:** Federica Sancassiani, Alessandra Perra, Alessia Galetti, Lorenzo Di Natale, Valerio De Lorenzo, Stefano Lorrai, Goce Kalcev, Elisa Pintus, Elisa Cantone, Marcello Nonnis, Antonio Egidio Nardi, Roberta Montisci, Diego Primavera

**Affiliations:** 1 Department of Medical Sciences and Public Health, University of Cagliari, 09124 Cagliari, Italy; federica.sancassiani@unica.it (F.S.); galettialessia@gmail.com (A.G.); stefanolorrai@live.it (S.L.); pintuselisa@yahoo.it (E.P.); elisa.cantone@libero.it (E.C.); rmontisc@gmail.com (R.M.); diego.primavera@tiscali.it (D.P.); 2 IDEGO Digital Psychology Society, 00197 Rome, Italy; lorenzodinatale86@gmail.com; 3 CEREBRUM VR, 00197 Rome, Italy; valerio.delorenzo@gmail.com; 4 Department of Systems Medicine, University of Tor Vergata, 00133 Rome, Italy; 5 The National Alliance for Neuromuscular Diseases and Neuroscience GANGLION Skopje, Skopje 1000, North Macedonia; gocekalcev@yahoo.com; 6 Department of Pedagogy, Psychology, Philosophy, University of Cagliari, 09123 Cagliari, Italy; marcello.nonnis@unica.it; 7 Institute of Psychiatry, Federal University of Rio de Janeiro, Rio de Janeiro 21941-901, Brazil; antonioenardi@gmail.com

**Keywords:** alexithymia, bipolar disorder, COVID-19, cognitive remediation, virtual reality, advanced technology laboratory

## Abstract

**Background:** Alexithymia, a predictor in chronic illnesses, like cardiovascular and bipolar disorder (CD–BD), could be improved with a virtual reality (VR) cognitive remediation program. This secondary analysis of a previous randomized controlled trial (RCT) evaluates alexithymia improvement and its factors in an experimental group versus a control group, exploring extensions to individuals with comorbid non-psychiatric chronic conditions. **Methods:** A feasibility cross-over RCT (ClinicalTrials.gov NCT05070065) enrolled individuals aged 18–75 with mood disorders (BD, DSM-IV), excluding those with relapses, epilepsy, or severe eye conditions due to potential risks with VR. Alexithymia levels were measured using the Toronto Alexithymia Scale with 20 items (TAS-20). **Results:** The study included 39 individuals in the experimental group and 25 in the control group, with no significant age or sex differences observed. Significantly improved alexithymia scores were noted in the experimental group compared to controls (F = 111.9; *p <* 0.0001) and in subgroups with chronic non-psychiatric comorbidities (F = 4.293, *p* = 0.048). Scores were particularly improved for difficulty in identifying feelings (F = 92.42; *p <* 0.00001), communicating feelings (F = 61.34; *p <* 0.00001), and externally oriented thinking (F = 173.12; *p <* 0.00001). **Conclusions:** The findings highlight alexithymia enhancement in BD, even with comorbid non-psychiatric chronic diseases. Given its impact on BD progression and related conditions, like CD, developing and evaluating VR-based tools in this context is suggested by these findings.

## 1. Background

The term “Alexithymia” originates from the Greek words “lexis” (word) and “thymos” (emotion), coined by A (the Greek privative), thus signifying “lack of words for emotions”. Peter Emanuel Sifneos introduced this term to delineate the characteristics observed in individuals exhibiting psychosomatic symptoms who struggled to engage in a psychotherapeutic relationship [1,2]. Those with high levels of alexithymia exhibit minimal awareness of their emotional experiences and symptoms, along with difficulties in expressing their moods and feelings, and struggle to emotionally engage with situations. When questioned about their emotional state, they tend to describe their discomfort in vague and confusing terms, often attributing it to bodily sensations rather than psychological distress [3]. Therefore, alexithymia is characterized as a personality trait marked by (a) difficulty in distinguishing and identifying specific emotions, including discerning somatic sensations from emotions; (b) significant difficulty in expressing one’s emotions verbally; (c) limited imaginative capacity; and (d) a tendency to focus on external stimuli with a lack of attention towards internal cues [1,2,3,4,5,6]. Alexithymia has been linked to numerous chronic illnesses [7,8] and is recognized as a negative prognostic factor, particularly in cardiovascular disorders (CDs), which are closely associated with bipolar disorder (BD) [9,10]. Actually, it plays a significant role in delaying treatment for acute myocardial infarction [11], consequently impacting patient outcomes. Following a myocardial infarction, it is linked to lower survival rates [12]. Only recently has research focused to the significance of alexithymia in BD. It is now evident that individuals with this disorder typically exhibit higher levels of alexithymia compared to those without it [13,14]. Furthermore, there is an observed association between high level of alexithymia and histories of childhood abuse, as well as an elevated risk of suicide among individuals with BD. Specifically, emotional dysregulation appears to act as a mediating factor in the risk of suicidal ideation [15]. Therefore, it is not surprising that alexithymia is recognized as a predictor of adverse outcomes and social decline in individuals with BD [16,17]. To address these complex needs, mental health rehabilitation aims to employ intervention models that integrate diverse techniques in accordance with theoretical frameworks and evidence-based practices [18,19,20]. New evolutionary models, which consider the social determinants of health, interpret illnesses not merely as products of biological determinism but rather as outcomes influenced by multiple factors that contribute to the development of both physical and mental comorbidities [21,22,23]. These factors also limit individuals in their achievement of recovery goals, including personal and social aspects [21]. Thus, it is crucial for rehabilitative interventions to be recovery-oriented and to incorporate these multifactorial aspects into their methodology [24]. In order to attain their subjective recovery goals, it becomes of primary importance to develop tools capable of modifying also alexithymia levels in individuals suffering from both BD and CD.

In this regard, cognitive remediation (CR) interventions are currently recognized as a promising treatment for BD [25,26,27,28,29]. They offer the potential to bridge the gap between symptomatic relief and achieving full functional recovery by improving not only cognitive outcomes but also clinical and social functioning [25,30,31]. This is due to the intervention method incorporating combined cognitive and behavioral techniques aimed at generalization into everyday life [32,33]. With the advancement of technology and its application in prevention, treatment, and rehabilitation settings, CR training are also integrating technologies such as virtual reality (VR) [34,35]. Fully immersive VR offers the intrinsic advantage of rendering the rehabilitation setting experience appealing, aligned with the individual’s real-life needs, and facilitating learning [36,37,38,39,40,41].

For these reasons, immersive VR-based CR training has been developed for BD treatment, aiming not only to improve cognitive processes but also addressing alexithymia, biological and social rhythms, quality of life, and depression and anxiety symptoms. A comprehensive and multidimensional approach is required, ranging from cognitive and emotional aspects to rhythm regulation and symptom management, components primarily implicated in BD. The current study is part of a previous randomized controlled trial (RCT) that evaluated the feasibility of the intervention in this specific population as the primary outcome, and the preliminary efficacy regarding cognitive, clinical and functional variables as secondary outcomes; the protocol and the results were previously published [42,43].

## 2. Aims

The aim of this research is to conduct a detailed secondary analysis of the previously mentioned randomized controlled trial (RCT), investigating the preliminary efficacy of VR-based cognitive remediation (VR-CR) training (NCT05070065). The initial study observed an enhancement in alexithymia levels, evidenced by a reduction in scores on the Toronto Alexithymia Scale with 20 items (TAS-20) [44] linked to the VR intervention [42]. This secondary analysis seeks to achieve several objectives: firstly, to understand the change and quantify the degree of improvement in alexithymia levels in the VR-treated group compared to the control group; secondly, we aimed to identify which specific aspects of alexithymia (difficulty recognizing feelings, difficulty describing feelings, and externally oriented thinking) are most influenced by the intervention; and thirdly, to determine if the observed improvement in alexithymia extends to the subgroup of participants with mood disorders (specifically BD, as defined by *Diagnostic and Statistical Manual of Mental Disorders*, *Fourth Edition*) who also have comorbid non-psychiatric chronic diseases.

By addressing these objectives, this study aims to provide a more comprehensive understanding of the future efficacy of VR-CR training in improving emotional processing difficulties. The findings will contribute to the broader field of psychosomatic medicine by highlighting the potential of VR-based interventions to enhance emotional awareness and regulation in individuals with complex clinical profiles.

## 3. Methods

### 3.1. Study Design

This study is a feasibility cross-over RCT [42], with the protocol number NCT05070065 registered on ClinicalTrials.gov in 2021. The primary aim of the original investigation was to evaluate the feasibility of the study, while the current analysis focuses on one of the declared secondary outcomes, namely the “level of alexithymia” [43]. The design and execution of the survey adhered strictly to the CONSORT guidelines for feasibility studies, ensuring methodological rigor and transparency [44]. The primary outcome of the initial study was to assess various aspects of feasibility, including participant recruitment, retention rates, and adherence to the intervention protocol and clinical outcomes as secondary outcomes. For the current study the secondary analysis presented here specifically examines changes in alexithymia levels among participants, providing additional insights into the potential benefits of the intervention beyond the primary feasibility outcomes. This comprehensive approach allows for an understanding of both the practicality of implementing the intervention and its impact on emotional processing difficulties, which is crucial for informing future research and clinical practice.

### 3.2. Sample

Participants enrolled in the randomized controlled trial (RCT) were diagnosed with a mood disorder, specifically bipolar disorder (BD), according to the diagnostic criteria outlined in the American Psychiatric Association’s *Diagnostic and Statistical Manual of Mental Disorders, Fourth Edition* (DSM-IV) [45]. They were recruited from individuals seeking treatment at the Consultation Psychiatry and Psychosomatic Center of the Hospital “San Giovanni di Dio” (University Hospital of Cagliari).

Inclusion criteria required individuals to be aged between 18 and 75 years old, with no gender restrictions, and to have provided informed consent before the intervention began. Exclusion criteria comprised current manic or depressive episodes due to the severity of symptoms, which would preclude bi-weekly participation over three months in an outpatient setting, and severe eye diseases or epilepsy due to the potential exacerbation or recurrence of symptoms under VR stimulation. Participants were randomized into two groups by a biometrician utilizing a computerized algorithm, ensuring blinding with respect to the final participant identifiers. The researcher overseeing the randomization process was not involved in the subsequent phases of the study. Additionally, the researchers responsible for psychometric assessments were blinded to the specific intervention assigned to each participant, ensuring the integrity and objectivity of the assessment process. This rigorous methodology was designed to minimize bias and enhance the reliability of the study’s findings.

### 3.3. Experimental and Control Groups

Participants assigned to the experimental group received fully immersive VR-CR training. The VR environment was generated by the “CEREBRUM” software (version 3.0.1), developed by the Italian small and medium enterprise Cerebrum VR (Italy). A comprehensive description of the software is available in previously published papers [42,43]. In summary the device used is an all-in-one headset Oculus Go, CE-marked, providing 360° visual immersion, with the use of a controller to select options during the exercises. The immersive scenarios encompassed domestic and urban settings, directly facilitating training of both cognitive and personally functional strategies through everyday life tasks. During the exercises, auditory stimuli are also present, including exercise instructions, urban sounds, weather-related sounds, and in some scenarios, social settings with dialogue.

The tasks varied in difficulty, allowing clinicians to adjust the exercises based on participants’ functional performance levels and specific competencies. Consequently, the learning process was stimulating rather than frustrating, as the exercises were adapted to each participant’s specific level. The intervention consisted of 24 sessions, each lasting 45 min, conducted twice a week, and completed over 3 months. It was manualized and developed with a human-centered [46,47], recovery-oriented, and bio–psycho–social approach [24,48,49]. The session methodology was specifically described in the trial protocol [43]. In summary, it adheres to an integrated hierarchical approach where emphasis is placed on training strategies that integrate emotional, cognitive, metacognition, social cognition and behavioral techniques [20,32,50]. Indeed, each session included an introduction with mindfulness techniques to promote emotional integration in the cognitive learning process, psychoeducation on functions to enhance awareness and self-monitoring, and generalization to promote the implementation of trained strategies in the individual’s life context according to their needs. Both the control group and the experimental group received treatment as usual (psychiatric consultation with pharmacotherapy with or without psychotherapy). Therefore, the comparison is between the experimental group receiving VR plus treatment as usual versus the control group receiving only treatment as usual. The intervention was carried out by mental health rehabilitation experts, including psychiatric rehabilitation technicians and psychologists [51].

### 3.4. Outcome and Study Tools

The outcome of this secondary analysis is the level of alexithymia, as measured by the TAS-20 [6,52] in its Italian version [53]. Originally, in the research protocol registered on ClinicalTrials and approved by the local Ethics Committee (see the Ethical Aspects section), this was designated as a secondary outcome. The TAS-20 is a self-administered questionnaire consisting of 20 items, each rated on a Likert scale from 1 (strongly disagree) to 5 (strongly agree). The TAS-20 provides a total score that reflects the overall level of alexithymia. Additionally, the tool assesses three primary dimensions central to the construct of alexithymia: (1) difficulty recognizing feelings and distinguishing them from physical sensations (F1), (2) difficulty describing and communicating one’s feelings to others (F2), and (3) a cognitive style oriented toward external realities (F3). The TAS-20 has demonstrated good reliability, as well as factorial, convergent, and discriminant validity. Scores range from 20 to 100, with a total score exceeding 60 indicating the presence of significant alexithymia. This comprehensive tool allows for a nuanced understanding of the various facets of alexithymia, making it a valuable instrument in the assessment of emotional processing difficulties [6,52].

### 3.5. Statistical Analysis

The change in the TAS-20 total score (in the overall sample experimental and control sample and in subsamples of people with comorbid chronic diseases) and of each TAS-20 sub scale within group by time (To vs T1) was calculated as difference in the mean score ± standard deviation (SD); the differences in score change T0 vs T1 between groups was measured using ANOVA one-way statistics. The comparison of non-parametric variables was carried out using the chi-square test with Yates correction where needed. A *p* value *<* 0.01 was considered statistically significant. The analysis of the change in the frequency of people with alexithymia in the groups from T0 to T1 was carried out by using the analysis of variance for nominal data by Castellan [53].

## 4. Results

The final experimental sample consisted of 39 individuals out of the initially recruited 50, with some dropouts occurring during follow-up. This was compared to 25 individuals in the control group, and no dropouts occurred. The degrees of freedom correspond to df = 1.62.

No statistically significant differences were observed between the experimental and control groups in terms of mean age and frequency of sex, as shown in Table 1, both in the total groups and in subgroups with comorbid chronic diseases. Within the subgroup of individuals with comorbid chronic diseases, the experimental group included seven individuals with chronic thyroid disease, four with CD, three with neurological disorders, three with autoimmune or rheumatological diseases, three with female genital disorders, one with diabetes, two with spinal hernias, and two with chronic gastrointestinal diseases (some individuals experienced multiple disorders). In contrast, the control group comprised two individuals with chronic thyroid disease, one with CD, one with neurological disorders, two with autoimmune or rheumatological diseases, three with female genital disorders, one with diabetes, and two with chronic gastrointestinal diseases (some individuals experienced multiple disorders). As showed in Table 2, a notable decrease was observed in the overall TAS-20 score, indicating an improvement in alexithymia within the experimental group compared to the control group. This trend was also evident across the three components, namely difficulty identifying feelings, difficulty communicating feelings to others, and externally oriented thinking. Similarly, within the experimental group, there was a decrease in the frequency of individuals with high alexithymia, dropping from 43.5% at T0 to 25.6% at T1. In contrast, the control group witnessed an increase in individuals with high alexithymia, rising from 24% to 52% (analysis of variance for nominal data by Castellan), as detailed in Table 3. The contrast in alexithymia improvement from T0 to T1 persists when comparing the two subgroups, namely experimental and control, that presented comorbidity with another chronic non-psychiatric pathology (Table 4).

## 5. Discussion

The study highlighted that fully immersive VR-based CR training for individuals with BD could led to improvements in alexithymia, as assessed by the TAS-20 scale, within the experimental group compared to the control group and possible prevention of worsening. Notably, there were significant enhancements in the scores of the three subscales constituting the construct of alexithymia when compared with the worsening in the control condition.

In line with these findings, the experimental group experienced a reduction in the number of individuals with high alexithymia at T1, whereas the control group did not exhibit such an improvement; on the contrary, they exhibited a worsening. Moreover, the positive impact on alexithymia following the intervention was evident when comparing subgroups with or without chronic non-psychiatric pathologies. This finding holds significance considering that alexithymia has been identified as a negative prognostic factor in BD. While the direct correlation between improving alexithymia and the course of BD remains uncertain, it is plausible that such improvement could positively influence the disorder; certainly, these interpretations (which provide no information regarding the correlation) must be confirmed in future studies. This assumption is rooted in the recognition that alexithymia indicates poor awareness of one’s emotional experiences. In the management of BD, early symptom recognition and communication are essential for relapse prevention [54,55]. Several pieces of evidence support the notion that early symptom recognition is integral to psychosocial interventions aimed at preventing relapse [56,57,58,59,60]. Recent research has highlighted the presence of individuals exhibiting hyperactivity traits who function well socially and professionally without evident pathologies, despite representing a potential area of vulnerability towards BD, particularly under stressful conditions [60,61,62]. Developing an effective tool to address alexithymia could prove invaluable for such individuals, especially during periods of heightened stress that may trigger BD. In hyperactive individuals, there might be a tendency to deny symptoms, potentially leading to maladaptive coping mechanisms [63,64,65,66].

Another area of potential interest lies in the prevention of chronic pathologies, with particular attention to certain conditions, like CD. Our study reveals an improvement in alexithymia in the experimental group when compared with the worsening in the control condition, even in cases of BD comorbid with chronic non-psychiatric conditions. This suggests that it could be valuable to investigate whether a similar outcome is achievable in individuals with CD, specifically those with cardiovascular atherosclerosis and post-myocardial infarction patients. As noted in the introduction, alexithymia is a significant barrier to seeking treatment among individuals who have experienced a heart attack [3,11]. Additionally, alexithymia is associated with increased mortality following a heart attack. Difficulty in interpreting bodily symptoms may lead to delayed treatment-seeking behavior. Therefore, an intervention that is both enjoyable and easy to implement, with a recovery-oriented approach, could serve as a valuable preventive tool for at-risk populations. Employing a comprehensive intervention methodology, which addresses various needs and is translated into multidimensional techniques, could serve as a reproducible and effective approach for managing comorbidity across different disorders. For instance, it could be used to manage the relationship between BD and CD [67], as the latter constitute one of the leading causes of premature death in individuals with BD [68,69,70].

In conclusion, it is essential to explore why the intervention achieved this important and somewhat unexpected outcome. One hypothesis for future research is whether the effect on alexithymia could be a secondary result of cognitive improvement. However, a crucial factor may be the mindfulness component integrated into each session, along with the VR experience that fosters a sense of a “judgment-free” environment. Indeed, a central aspect of mindfulness practice is cultivating an open, non-reactive awareness of inner experiences, which is vital for emotional regulation [71].

### 5.1. Limitations

The main limitations of the present study are related to the sample size and the absence of an intention-to-treat analysis. Specifically, the sample size is insufficient to definitively demonstrate the effectiveness of these results. Additionally, the marked worsening observed in the control sample could be due to chance, and, therefore, this finding should be supported by more robust phase three studies.

### 5.2. Implication for Research

Future prospective research should investigate whether similar outcomes can be achieved in individuals with chronic diseases, particularly those with cardiovascular atherosclerosis and post-myocardial infarction patients. Furthermore, exploring whether alexithymia could be a secondary outcome of cognitive improvement, especially when combined with mindfulness, in the treatment of BD. It is also crucial that these findings be validated in studies with larger sample sizes capable of demonstrating their effectiveness.

## 6. Conclusions

This study demonstrates an improvement in alexithymia, when compared with the worsening in the control condition, among individuals with BD, even when they are also managing other non-psychiatric chronic diseases, and, thus, are presenting with comorbid conditions. The improvement was achieved through a VR-based CR intervention utilizing a multidisciplinary approach, including mindfulness techniques, cognitive training in VR, and the generalization of trained strategies for daily life. The study primarily targeted other aspects of BD, such as feasibility outcomes and cognitive impairment. Given the significant role that alexithymia plays in the chronic course of BD and other chronic conditions, such as CD, our findings underscore the importance of developing and rigorously studying the efficacy of specialized tools. These tools should utilize fully immersive VR technology and be oriented towards recovery, aiming to address not only the primary symptoms of BD but also the comorbid conditions that often accompany it.

## Figures and Tables

**Table 1:** Study sample and subgroups with chronic non psychiatric disease.

	Experimental Group	Control Group	Statistics
Sex (female)	14 (64.1%)	7 (72%)	Chi square 1 df = 0.431; *p* = 0.512
Age	47.51 ± 13.52	46.28 ± 13.40	F 1.63 df = 0.127; *p* = 0.723
Subgroup with chronic diseases (Sex—female)	15 (78.9%)	8 (80%)	Chi square Yates Correction 1 df = 0.001; *p* = 0.999
Subgroup with chronic diseases (age)	52.26 ± 12.89	49.8 ± 12.84	

**Table 2:** Tas-20 global and sub-scales scores comparison within group by time (To vs. T1) with mean difference ± standard deviation and one way-repeated measures ANOVA (F, *p* value).

		T0 Mean ± SD	T1 Mean ± SD	T0 × T1 Mean Difference ± SD
**Tas Global**	EX (N = 39)	55.12 ± 14.51	49.66 ± 14.87	−5.46 ± 2.06
	CC (N = 25)	52.08 ± 14.52	55.76 ± 16.13	+3.68 ± 2.36
				F = 267.5; *p <* 0.00001
**F1—**Difficulty identifying feelings	EX (N = 39)	20.84 ± 7.29	18.05 ± 8.43	−2.27 ± 1.25
	CC (N = 25)	19.87 ± 78.37	20.44 ± 7.58	+0.57 ± 0.98
				F = 92.42; *p <* 0.00001
**F2—**Difficulty communicating feelings to others	EX (N = 39)	15.23 ± 4.77	14.35 ± 4.74	−0.88 ± 0.54
	CC (N = 25)	14.56 ± 4.85	15.00 ± 5.23	+0.44 ± 0.81
				F = 61.34; *p <* 0.00001
**F3—**Externally oriented thinking	EX (N = 39)	19.05 ± 5.88	17.66 ± 4.69	−1.39 ± 1.16
	CC (N = 25)	17.72 ± 4.50	20.32 ± 6.33	+2.60 ± 1.22
				F = 173.12; *p <* 0.00001

**Table 3:** Change in frequency from T0 to T1 of individuals with high alexithymia (Tas-20 score ≥ 60) by time and group.

	T0	T1	Analyis of Variance for Nominal Data	*p*
Experimental group	17/39 (43.5%)	10/39 (25.6%)	143.19	*p <* 0.0001
Control group	6/25 (24%)	13/25 (52%)		

**Table 4:** TAS-20 Subgroups with comorbid chronic diseases including cardiovascular.

	T0	T1	T0×T1 Mean Difference ± SD
Experimental subgroup with chronic diseases T0 (N = 19)	55.57 ± 15.58	56.47 ± 14.61	−1.10 ± 2.01
Control subgroup with chronic diseases T0 (N = 10)	57.5 ± 14.41	58.10 ± 15.27	+0.60 ± 2.27
			F = 4.293 *p* = 0.048

## Data Availability

Data are contained within the article.
